# A Protein Co-Conservation Network Model Characterizes Mutation Effects on SARS-CoV-2 Spike Protein

**DOI:** 10.3390/ijms24043255

**Published:** 2023-02-07

**Authors:** Lianjie Zeng, Yitan Lu, Wenying Yan, Yang Yang

**Affiliations:** 1 School of Computer Science & Technology, Soochow University, Suzhou 215000, China; 2 Collaborative Innovation Center of Novel Software Technology and Industrialization, Nanjing 210000, China; 3 Department of Bioinformatics, School of Biology and Basic Medical Sciences, Medical College of Soochow University, Suzhou 215123, China; 4 Jiangsu Province Engineering Research Center of Precision Diagnostics and Therapeutics Development, Suzhou 215123, China

**Keywords:** COVID-19, conservation, co-conservation, protein co-conservation weighted network, spike protein

## Abstract

The emergence of numerous variants of SARS-CoV-2 has presented challenges to the global efforts to control the COVID-19 pandemic. The major mutation is in the SARS-CoV-2 viral envelope spike protein that is responsible for virus attachment to the host, and is the main target for host antibodies. It is critically important to study the biological effects of the mutations to understand the mechanisms of how mutations alter viral functions. Here, we propose a protein co-conservation weighted network (PCCN) model only based on the protein sequence to characterize the mutation sites by topological features and to investigate the mutation effects on the spike protein from a network view. Frist, we found that the mutation sites on the spike protein had significantly larger centrality than the non-mutation sites. Second, the stability changes and binding free energy changes in the mutation sites were positively significantly correlated with their neighbors’ degree and the shortest path length separately. The results indicate that our PCCN model provides new insights into mutations on spike proteins and reflects the mutation effects on protein function alternations.

## 1. Introduction

Coronavirus disease 2019 (COVID-19), caused by severe acute respiratory syndrome coronavirus 2 (SARS-CoV-2), has posed a serious global public-health emergency and was declared as a global pandemic by the World Health Organization (WHO) on 11 March 2020. The infection possesses a clustering onset and targets the respiratory framework of its host, causing influenza-like sickness with symptoms such as cough, fever, and then, in progressively serious cases, troubled breathing [1,2,3]. COVID-19 has spread rapidly over 200 countries and territories around the world, which has resulted in more than 661 million coronavirus cases and six million deaths (https://www.worldometers.info/coronavirus/, accessed on 24 December 2022).

SARS-CoV-2 has been confirmed to enter the host cells by contact between its receptor-binding domain (RBD) of the spike (S) protein and angiotensin converting enzyme 2 (ACE2) of the host cell [4,5]. Although substantial progress in clinical research has led to a better understanding of SARS-CoV-2 and the management of COVID-19, many countries have endured several waves of outbreaks due to the emergence of variants [6]. The *S* protein displays a high degree of genetic variability in the virus genome [7], and its variants make additional close contacts with ACE2, correlating with higher binding affinity and perhaps increased infectivity [8]. For example, the Omicron variant (B.1.1.529) can evade immunity from natural infection or vaccines and increase the risk of reinfections [9,10,11]. Acquiring a comprehensive knowledge of the S protein variants is critical for the determination of a defensive approach against COVID-19.

The biological network approach, which is based on local and global topological features of the network, has been extensively used in various biological fields [12], and has also been applied in COIVD-19 related research. Deisy constructed a network medicine framework to identify potentially effective drug candidates [13]. Luisa et al. applied protein contact networks and found that the allosteric modulation region modulates the binding of the spike protein with ACE2 in different conformational states of the spike protein [7,14]. One of the main network-based strategies is to convert a protein into a network to study the structure, function, and dynamics of proteins [15,16,17]. However, most of the network models for protein function study require the 3D structures of proteins that can be experimentally determined, but the process is still laborious and time-consuming. In particular, the emergence of new variants of COVID-19 brings challenges for structure-based network models if the structures from the variants are not determined.

**Figure 1:**
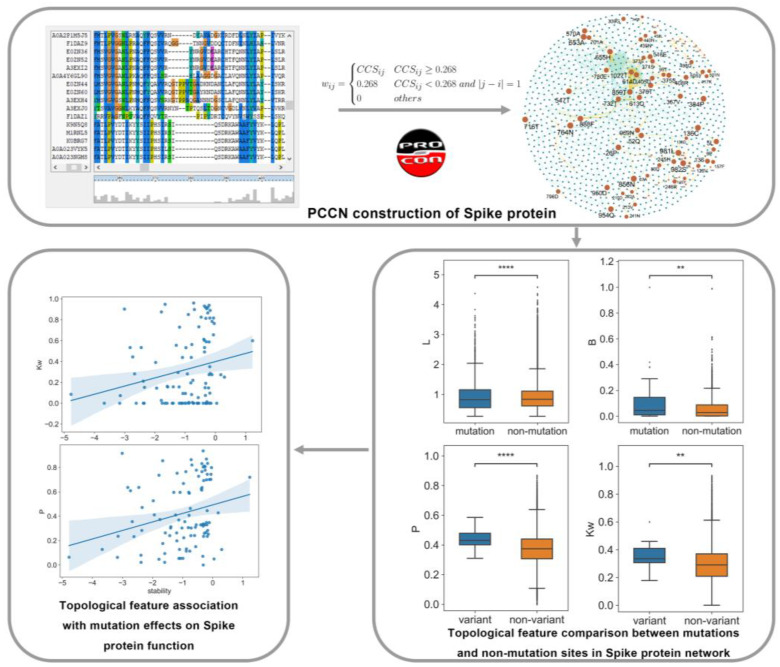
Overview of our method to study the mutation effects on the spike protein. ** *p* < 0.01; **** *p* < 0.0001.

In this study, we propose a protein co-conservation weighted network (PCCN) model simply based on protein sequence to investigate the mutation effects on the spike protein (Figure 1). We then compared the network topological features of the mutation and non-mutation sites at the residue and variant levels. Finally, we mined the correlation of topological features with the mutation effects on the spike protein stability changes and binding free energy changes.

## 2. Results

### 2.1. Conservation and Co-Conservation of the Spike Protein

To calculate the conservation of the spike protein, the protein family was initially searched in InterPro [18] for the Spike glycoprotein, beta-coronavirus (IPR042578), and 11,372 related sequences were found. Among these sequences, those within the same species had high similarity, so only one of them was retained, leaving 263 sequences that were aligned to calculate the conservation (*CS*) and co-conservation (*CCS*) using ProCon [19,20].

**Figure 2:**
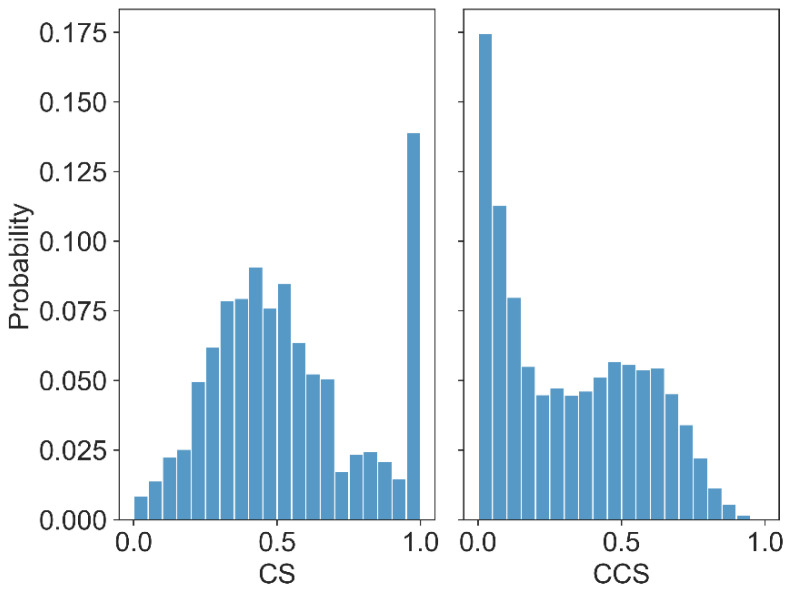
The distribution of the residue conservation scores in the spike protein.

The distribution of *CS* is shown in Figure 2. The left panel shows the conservation of each residue, which shows a trend of normal distribution. Notably, the scores in the range of 0.9 to 1.0 were particularly high. On the right, the *CCS* among residues is shown, which followed an overall trend of normal distribution, except that the *CCS* was small. The results demonstrated that the rate of residues with high conservation residues and low co-conservation was relatively high, which indicated that many of the residues in the spike protein were conserved, but their co-conservation was relatively weak.

**Figure 3:**
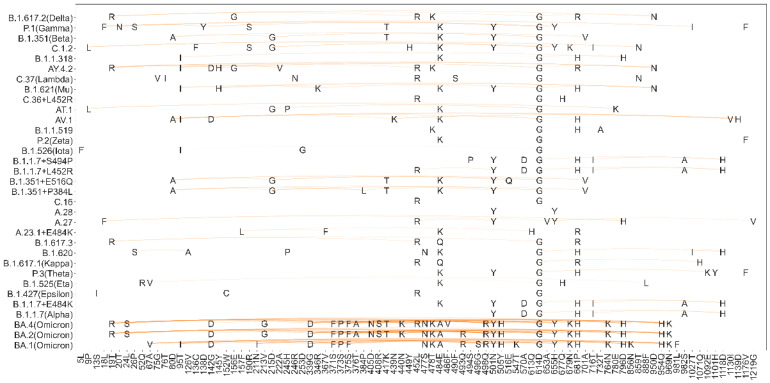
The co-conservation of mutation sites in popular variants.

Next, an investigation into the conservation and co-conservation of mutation sites in variants that indicate a significant impact on transmissible, severity, and immunity was conducted. As shown in Figure 3, most variants had substitutions of D614G, followed by substitutions at sites 484E, 681P and 501N, which are relatively common and considered to increase infectivity with clear evidence [21,22,23,24,25,26,27,28,29,30]. Regarding the co-conservation of mutation sites in the variants, most of the variants had strong co-conservation site pairs, which may indicate that the strong co-conservation sites may play an important role in infectiousness. However, we also found that although some sites (such as 484E, 681P, and 417K) appeared more frequently, they did not have strong co-conservation with other sites.

### 2.2. Protein Co-Conservation Network Construction

A protein co-conservation weighted network of spike protein was constructed based on the *CCS* score that was calculated from the sequence of the protein. As shown in Figure 1, there were 1273 amino acids in the spike protein, which corresponded to 1273 nodes in the network. A total of 264,046 (16.3%) of all possible residue pairs had strong co-conservation as edges in the network, and the weights of the edges were *CCS*. Thus, the PCCN model could reveal the co-conservation information of residues from a network view.

### 2.3. Network Topological Features of PCCN of Spike Protein

Next, the topological features of PCCN were investigated and the correlation between these network features and the infectious or pathogenicity of SARS-CoV-2 was further explored. The network analysis was performed at two levels: residues and variants.

#### 2.3.1. Mutation Sites Showed High Centrality in PCCN at the Residue Level

**Figure 4:**
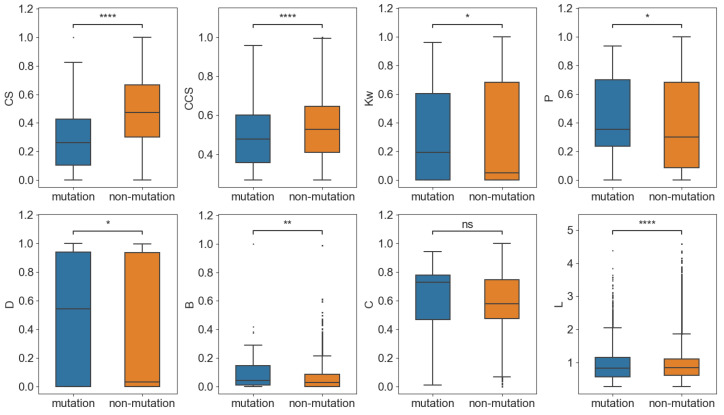
Comparison of the network features of the mutation and non-mutation sites. * *p* < 0.05; ** *p* < 0.01; **** *p* < 0.0001.

In the network, 89 nodes (residues) produced substitution mutations in the main variants. To compare the topological features of these 89 mutation nodes with the non-mutation nodes, we randomly selected 89 nodes from the non-mutation nodes and computed their average network parameters for 1000 times to present the network features of non-mutation nodes, which were then compared with the 89 mutation nodes. As shown in Figure 4, we calculated the average neighborhood weighted degree (*Kw*), page rank (*P*), degree centrality (*D*), betweenness (*B*), closeness (*C*), and shortest path length (*L*) and found that the mutation nodes had significantly larger *Kw*, *P*, *D*, *B*, but smaller *L* than the corresponding non-mutation nodes. The results indicate that the mutation nodes demonstrated more centrality than the non-mutation nodes and that they were topologically important in the PCCN. Moreover, the *CS* and *CCS* of the mutation nodes were significantly smaller than those of the non-mutation nodes, which showed that the conservation and co-conservation of the mutation nodes were relatively weak. Table S1 lists the detailed network feature information of the nodes.

**Figure 5:**
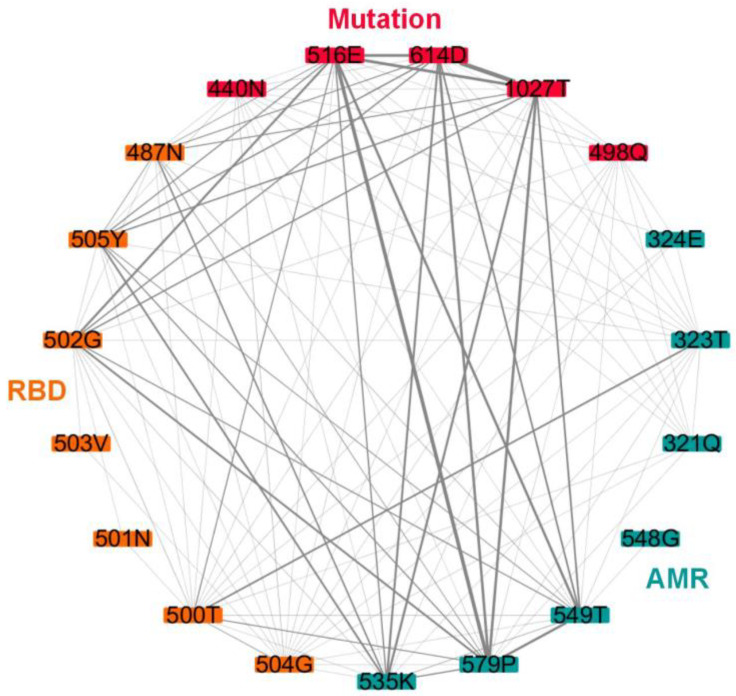
Interactions among top mutation sites with AMR and RBD. Red nodes: mutation sites; green nodes: AMR; orange nodes: RBD.

Finally, the top three mutation sites and co-mutation sites with the highest network parameters are listed in Table 1. Several substitutions had more than one high parameter such as 498Q, 440N, 516E, 1027T, and 614D. Then, we investigated these five substitution site relations with their allosteric modulation region (AMR) and RBD and found that 614D and 1027T had more interactions with both AMR and RBD (Figure 5). The results indicate that there was more of a co-conservation tendency among 614D, AMR, and RBD, which may be the reason for the mutation of 614D and will allow for a modulated structural rearrangement for membrane fusion [31] and increase the infectivity and stability of virions [25].

#### 2.3.2. Mutation Sites Showed High Centrality in PCCN at the Variant Level

In this element of the study, 33 special variants were collected that have caused great harm to human society and are considered as highly infectious or virulent. To study the variants from a network view, we compared the network features of variants with those of the non-variants. For the variant group, the average parameter of mutation sites in each variant was used to present the feature for the variant; therefore, there were 33 values in the variant group for each parameter. For the non-variant group, random selection was applied to achieve the same number of non-mutation sites as each variant and computed the average parameter 1000 times. For example, there were five substitutions in the Beta variant, and five nodes were taken from the non-mutation sites 1000 times. The distribution of network characteristics for the same number of nodes is shown in Supplementary Figures S1–S8.

**Figure 6:**
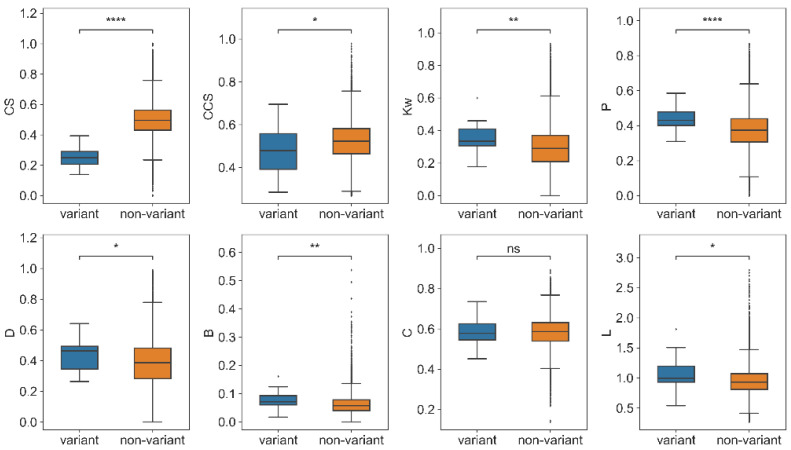
Comparison of the network features of the variant and non-variant groups. * *p* < 0.05; ** *p* < 0.01; **** *p* < 0.0001.

As a result, the variant group also showed significantly higher *Kw*, *P*, *D*, and *B* than the non-variant group (Figure 6). The results indicated that the mutation sites that occurred on the same variants have high centrality and may play an important role in their conservation. In addition, the *CS* and *CCS* of mutation nodes in the variants were significantly smaller than the non-mutation nodes in the non-variant group, which showed that the conservation and co-conservation of mutation nodes were relatively weak at the variant level.

**Figure 7:**
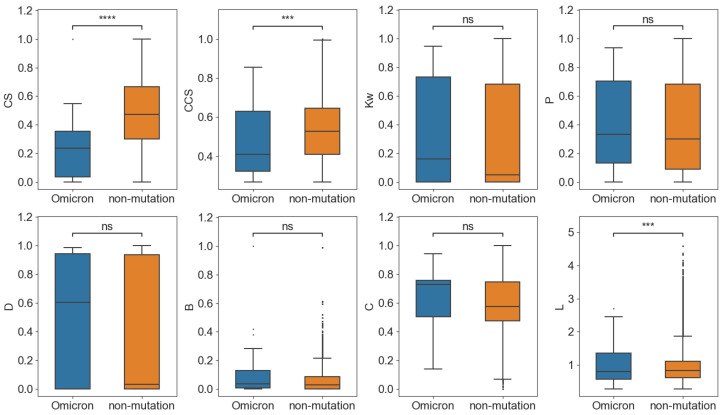
Comparison of the network features of Omicron BA.4 and the non-variant group. *** *p* < 0.001; **** *p* < 0.0001.

Finally, Omicron BA.4 was taken, which contained the most mutation sites among the variants, as an example to perform the network analysis. As shown in Figure 7, similar to previous results, the *CS* and *CCS* of the mutation sites in BA.4 were significantly smaller than the non-variant group, which means that both the conservation and co-conservation of the mutation sites in the variant were lower. For the network parameters, only the shortest path length in Omicron and the non-variant group showed significant differences.

### 2.4. Stability Changes of Spike Protein upon Mutations Significantly Correlated with the Node Page Rank and Neighbors’ Degree

To study the mutation effects for the spike protein function from a conservation network view, we performed the correlation analysis between the topological features of the mutation sites and mutation effects on the protein stability changes.

**Figure 8:**
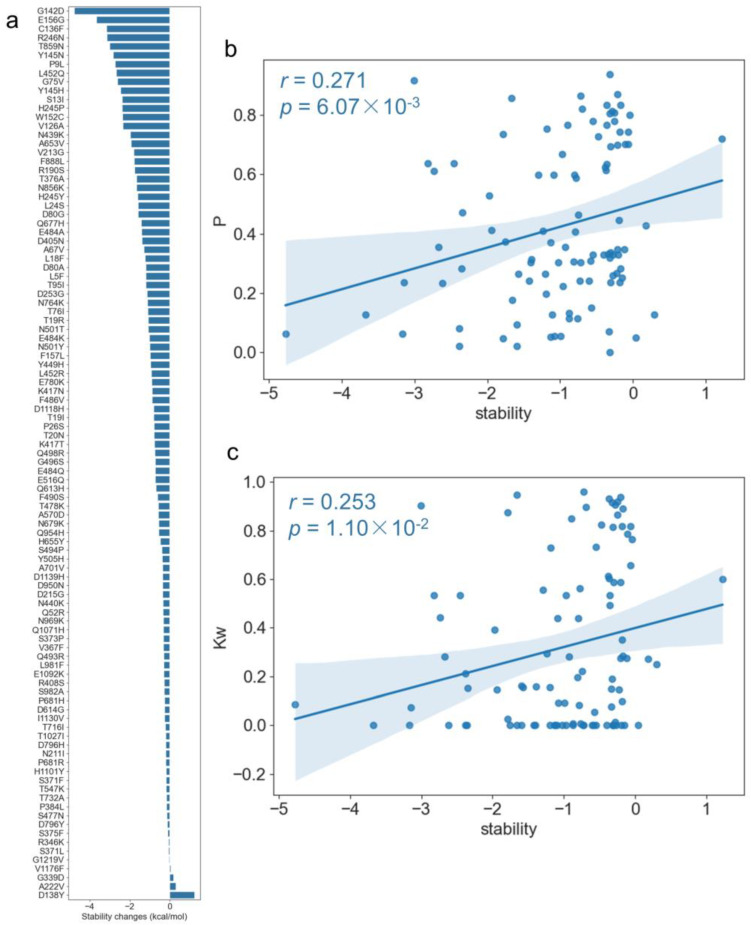
The correlation between the stability changes of the spike protein upon the mutations and PCCN topological features. (**a**). Stability changes in the spike protein upon the point mutations. (**b**). Page rank was positively significantly correlated with stability changes. (**c**). The average neighborhood weighted degree was positively significantly correlated with the stability changes.

DeepDDG [32] was used to predict the changes in stability for each point mutation and each mutation pair based on the spike protein structure. As shown in Figure 8a, most of the mutations were destabilizing mutations, for example, G142D, E156G, and C136F were the top three mutations that obtained the smallest stability scores, but D138Y obtained the largest positive stability score, which means that the mutation increased the stability. Then, we conducted a correlation analysis between the topological features and the stability changes upon mutations and found that three topological parameters including page rank, average neighborhood weighted degree, and degree centrality were significantly correlated with stability changes (Table S2). In particular, there was a positive correlation between the page rank (*P*) and stability changes with r = 0.271 and *p* = 6.07 × 10^−3^ (Figure 8b). A similar correlation pattern was also found between the average neighborhood weighted degree (*Kw*) and stability changes, as shown in Figure 8c (r = 0.253 and *p* = 1.10 × 10^−2^). The results indicate that if the nodes themselves had a high degree of centrality, high page rank, or their neighbors had a high degree, their mutations tended to destabilize the spike protein.

### 2.5. Binding Free Energy Changes of Spike Protein upon Mutations Significantly Correlated with CCS and Average Shortest Path Length

The binding of the spike protein with the human receptor ACE2 plays a central role in the molecular machinery of the SARS-CoV-2 infection of human cells. Mutations in the spike protein affect the binding affinity with ACE2 and have been established to be proportional to the infectivity of different viral variants in the host cells [4,33,34,35,36]. Therefore, we analyzed the relationship between the PCCN topological features and binding free energy (BFE) changes upon mutation to investigate the variant effects on protein binding from a network view based only on the sequence information.

**Figure 9:**
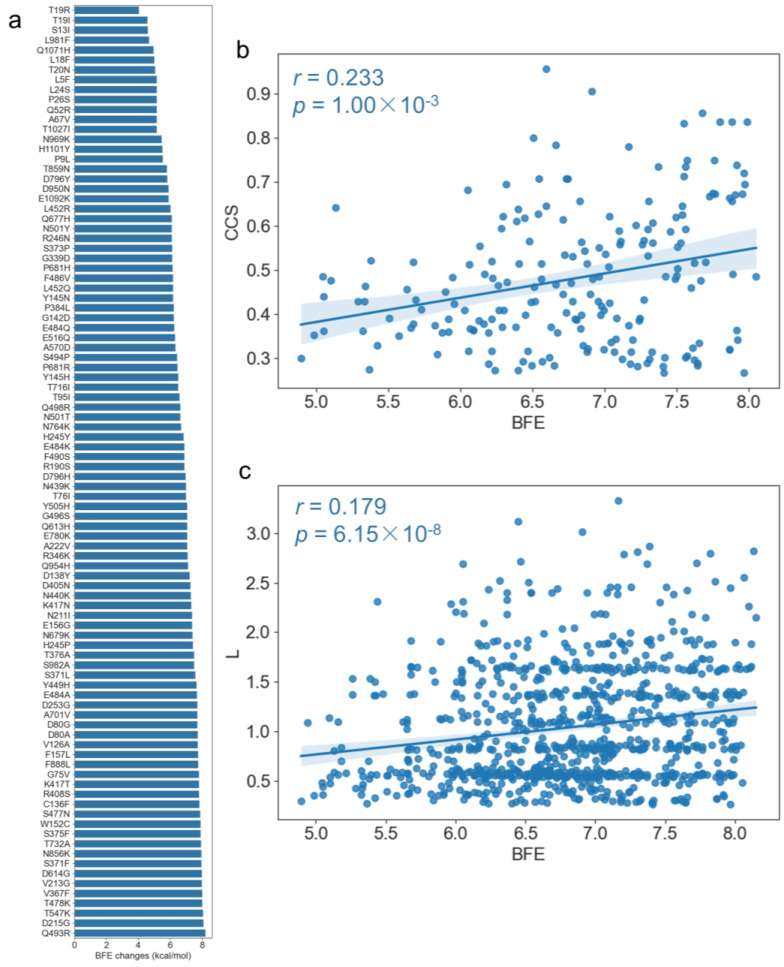
Correlation between the binding free energy changes of the spike protein upon mutations and the PCCN topological features. (**a**) Stability changes of the spike protein upon point mutations. (**b**) Page rank was positively significantly correlated with the stability changes. (**c**) BEF changes were positive significantly correlated with the average shortest path length *L*.

TopNetTree [37] was employed to estimate the BFE changes upon the mutations listed in Table 2 of the SARS-CoV-2 spike protein with ACE2 (PDB: 7A98 [38]). As shown in Figure 9a, all of the mutations increased the BFE between the spike glycoprotein and ACE2. The changes in BFE were significantly negatively correlated with the closeness centrality with the *p*-value of 0.045 and the coefficient of −0.207. Next, we analyzed the co-mutation effects on the spike protein. The correlation of the shortest path length and CCS between two mutations with the BFE changes of the two mutations were calculated, respectively. As shown in Figure 9b,c, the BFE changes were significantly positively correlated with CCS (r = 0.233 and *p* = 1.00 × 10^−3^) and shortest path length (r = 0.179 and *p* = 6.15 × 10^−8^) from the two mutation sites. The results demonstrate that if the two mutation sites have the tendency of high co-conservation or larger distance, they might induce larger BFE changes. Moreover, the top three co-mutation pairs with the largest BFE were D215G–D614G, S371F–D614G, and D614G–T732A. Notably, D614G was involved in all of them, which has been reported to have an important impact on infectivity [21,22,23,24,25,26,27]; although it is not in the RBD, it has an important impact on BFE. The results indicate that our PCCN model based on sequence information could also reflect the mutation effects on the binding affinity changes between the spike protein and ACE2. The detailed correlation parameters are listed in Table S3.

## 3. Discussion

Over the past years, the constant emergence of new variants of SARS-CoV-2 has presented challenges in the prevention of the spread and treatment of COVID-19. Studying the mutation effects on the spike protein from SARS-CoV-2 remains important. In this study, we proposed a sequence-based network model called PCCN that contains conservation and co-conservation information to study the mutation effects on the spike protein from a network view. We found that the mutation sites in the PCCN not only demonstrated lower conservation and co-conservation tendencies than the non-mutation sites, but also showed significant topological differences such as the average neighborhood weighted degree and page rank, both from non-mutation sites at the residue level and variant level. The results mean that the PCCN model could capture the topological features of mutation sites and thus has the potential to predict the mutation sites from non-mutation sites.

The stability and binding free energy of the spike protein were correlated with SARS-CoV-2 infectivity [4,33,34,35,36]. Thus, we analyzed the correlation between the topological features of PCCN and changes in the stability and binding free energy of spike protein upon mutations. The stability changes were significantly positively correlated with the node page rank and neighbors’ degree, while BFE changes were positively correlated with the shortest path length, and correlated negatively with the closeness centrality. The results indicate that our PCCN model could reflect the changes in the physico-chemical characteristics of the spike protein upon mutation.

However, this study had two potential limitations. First, the limited number of variants may result in bias for the topological feature comparison analysis between the variants and non-variants. Second, the relationship between the topological features and spike protein function needs further validation in large datasets or experimental verification. In future study, we will continue to collect the variants to explore and enhance the application of PCCN for the SARS-CoV-2 study.

Taken together, the sequence-based PCCN presented here not only suggests topological differences in mutation sites from non-mutations, but also provides new insights into the mutation effects on the spike protein physico-chemical characteristics without any protein structure information. Moreover, the PCCN was only based on the sequence information, which means that it can be quickly migrated to study the effects of continuously emerging variants of SASR-CoV-2. This is an adaptive way to face the emergence of new strains or viruses in the future.

## 4. Materials and Methods

### 4.1. Dataset

Since the outbreak of SARS-CoV-2, many variants have emerged on the spike protein that are highly infectious and have caused great destruction to the world such as Omicron [39,40], Beta [41,42,43,44], Gamma [44,45,46], and Delta [47]. In this paper, we focused on the variants provided by the ECDC. A total of 48 variants were collected from the ECDC website (https://www.ecdc.europa.eu/en/covid-19/variants-concern, accessed on 9 July 2022). The variants were divided into four types: variant of concern (VOC), variant of interest (VOI), variant under monitoring (VUM), and de-escalated variants (DEV). Then, 33 variants with currently available evidence of increasing transmissibility, immunity, or infection severity were kept for further analysis (Table 2). The substitutions of variants were selected from [48]. A total of 101 substitutions were observed that corresponded to 89 distinct mutation sites.

### 4.2. Conservation Calculation

The conservation score (*CS*) of each residue and the co-conservation score (*CCS*) between residues in the spike protein were calculated by ProCon [19,20], which is based on multiple sequence alignment (MSA) and information theory. The sequences for MSA were obtained from the InterPro database [18] by looking for its protein family to find similar sequences in different species. Then, sequences were aligned by ClustalX2 [49] and used as ProCon input to compute the spike protein conservation information.

The raw *CS* that was calculated by ProCon is between 0 and 3, and the *CCS* between residues is between 0 and 350. The scores were normalized by a min–max normalization method as shown in Equation (1), and they were mapped between 0 and 1:

(1) $$X=\frac{x-x_{min}}{x_{max}-x_{min}}$$

where $x_{max}$ and $x_{min}$ are the maximum and minimum scores, respectively.

Co-conservation scores for all possible residue pairs can be calculated by ProCon, and the larger the score, the stronger the co-conservation tendency. However, most of the residues pairs had weak co-conservation. We tried to keep more residue pairs with relatively strong co-conservation to construct the network. As shown in Figure 2, when the co-conservation score was smaller than 0.268, the number of residue pairs increased sharply, which means that they had less of a tendency of co-conservation. Therefore, residue pairs with *CCS* larger than 0.268 were selected as strong co-conservation.

In order to simplify the representation of the score, the *CS* and *CCS* denote the normalized conservation score and co-conservation score, respectively, in the later section.

### 4.3. Protein Co-Conservation Network Construction

The spike protein was represented as a network consisting of a set of nodes and edges. Each amino acid residue is presented as a node. The node set *V* is defined as follows:

(2) $$V=\{v_{i}|1<i<N\}$$

where *i* is the residue id in the protein; *v_i_* is the corresponding residue (node) for *I*; and *N* is the number of residues in the protein.

An edge was placed between two residues if the *CCS* between them was larger than 0.268. Thus, the adjacent matrix *W* of the PCCN was defined as follows:

(3) $$w_{ij}=\{\begin{array}{c} CCS_{ij}\;\;\;\;CCS_{ij}\;\geq 0.268\\ 0.268\;\;\;\;\;\;CCS_{ij}<0.268\;and\;\left|j-i\right|=1\\ \;\;\;\;0\;\;\;\;\;\;\;\;\;\;others\;\;\;\;\;\; \end{array}$$

When two nodes had strong co-conservation, that is, the co-conservation score was greater than or equal to the threshold of 0.268, there was an edge between the nodes and the weight of the edge was the *CCS*. The links between adjacent residues along the backbone were also kept and their weights were set at the threshold 0.268 if the *CCS* between them was less than the threshold. Thus, the PCCN is a network model that contains co-conservation information among residues.

### 4.4. Network Analysis

After constructing the PCCN, we analyzed the network topological characteristics of the residues in the network. Six network characteristics were used in our work: average neighborhood weighted degree (*Kw*), page rank (*P*) [50], closeness centrality (C), betweenness centrality (*B*), degree centrality (*D*), and shortest path length (*L*). For node *v_i_*, the parameters *Kw*, *D*, *B*, and *C* are defined as follows:

(4) $$Kw=\frac{\sum_{j\in \left\{j|w_{ij}\neq 0\right\}}w_{ij}}{n_{i}}$$

(5) $$D=\frac{n_{i}}{N-1}$$

(6) $$B=\frac{2}{\left(N-1\right)\left(N-2\right)}\underset{s,t\in V}{\sum }\frac{\sigma (s,t|v_{i})}{\sigma \left(s,t\right)}$$

(7) $$C=\frac{n_{i}-1}{N-1}\frac{n_{i}-1}{\sum_{j=1}^{n_{i}-1}\sigma \left(n_{i},\;n_{j}\right)}$$

where $n_{i}$ is the of adjacent nodes number of *v_i_*, and σ(s,t) is the shortest path length from node *s* to node *t*.

Page rank was originally designed to measure the importance of the website pages. Here, it was used to measure the importance of a node [50]. While it is an algorithm of directed graphs, each edge of the conservative interaction network will be converted to two directed edges. The shortest path length is the length of the path with the minimum sum of edge weights. Since all nodes are connected, there is an average shortest path between any pair. These network parameters were implemented by NetworkX [51].

### 4.5. Estimations of Binding Free Energy (BFE) Change

The change in binding free energy (*ΔΔG*) upon a single mutation from variants was estimated using TopNetTree [37]. The effects of two substitutions on the BFE changes were evaluated as follows:

(8) $$\Delta \Delta G_{ij}=\frac{\Delta \Delta G_{i}+{\Delta \Delta G}_{j}}{2}$$

where *ΔΔG_i_* and *ΔΔG_j_* are the binding free energy changes following mutation *i* and *j*, respectively; and *ΔΔG_ij_* is the combined changes of both mutations *i* and *j*.

### 4.6. Estimations of Stability Changes

The effect of the amino acid substitutions on stability was calculated using DeepDDG [32]. Similar to the binding free energy changes of two substitutions, the effects of two mutations on the stability changes were defined as follows:

(9) $$s_{ij}=\frac{s_{i}+s_{j}}{2}$$

where *S_i_* and *S_j_* are the stability changes following mutation *i* and *j* and *S_ij_* is the combined changes of stability following the mutation *i* and *j*. The structure of the spike protein was obtained from the website of Zhang’s laboratory (https://zhanggroup.org/COVID-19/index.html#table1, accessed on 30 October 2022) with the identification code QHD43416 that was generated by the D-I-TASSER/C-I-TASSER pipeline [52].

### 4.7. Statistics

The Spearman’s correlation coefficient was used to analyze the correlation between the protein properties and network characteristics. The Wilcoxon rank-sum test was applied to analyze the statistically significant differences and was implemented by SciPy [53].

## 5. Conclusions

In conclusion, we proposed a network model known as PCCN to study the mutation effects on the spike protein only from the sequence information. The topological features of PCCN showed a significant difference at both the residue and variant levels. In particular, the page rank and average neighborhood weighted degree could reflect the mutation effects on the spike protein stability, while the co-conservation score and shortest path length could indicate the mutation effects on the binding free energy changes. The PCCN model offers a new approach for studying variants that may cause future pandemics based on the sequence and topology information.

## Figures and Tables

**Table 1:** The top three substitutions and co-substitutions with the highest network characteristics.

Parameters	Top Three
*Kw*	516E, 376T, 1027T
*CS*	764N, 981L, 856N
*P*	969N, 859T, 1027T
*D*	95T, 516E, 215D
*B*	405D, 498Q, 440N
*C*	440N,498Q,1071Q
*CCS*	614D-1027T, 614D-859T, 376T-408R
*L*	190R-679N, 5L-253D, 190R-484E

**Table 2:** Summary of the 33 variants of the spike protein.

WHO Label	Lineage + Additional Mutations	Spike Mutations of Interest	Category	No. of Substitutions
Omicron	BA.1	A67V T95I G142D N211I G339D S371L S373P S375F S477N T478K E484A Q493R G496S Q498R N501Y Y505H T547K D614G H655Y N679K P681H N764K D796Y N856K Q954H N969K L981F	VOC	27
Omicron	BA.2	T19I L24S G142D V213G G339D S371F S373P S375F T376A D405N R408S K417N N440K S477N T478K E484A Q493R Q498R N501Y Y505H D614G H655Y N679K P681H N764K D796Y Q954H N969K	VOC	28
Omicron	BA.4	T19I L24S G142D V213G G339D S371F S373P S375F T376A D405N R408S K417N N440K L452R S477N T478K E484A F486V Q498R N501Y Y505H D614G H655Y N679K P681H N764K D796Y Q954H N969K	VOC	29
Alpha	B.1.1.7	N501Y A570D D614G P681H T716I S982A D1118H	DEV	7
n/a	B.1.1.7 + E484K	N501Y A570D D614G P681H T716I S982A D1118H E484K	DEV	8
Epsilon	B.1.427	S13I W152C L452R D614G	DEV	4
Eta	B.1.525	Q52R A67V E484K D614G Q677H F888L	DEV	6
Theta	P.3	E484K N501Y D614G P681H E1092K H1101Y V1176F	DEV	7
Kappa	B.1.617.1	L452R E484Q D614G P681R Q1071H	DEV	5
n/a	B.1.620	P26S V126A H245Y S477N E484K D614G P681H T1027I D1118H	DEV	9
n/a	B.1.617.3	T19R L452R E484Q D614G P681R	DEV	5
n/a	A.23.1+E484K	F157L V367F Q613H P681R E484K	DEV	5
n/a	A.27	L18F L452R N501Y A653V H655Y D796Y G1219V	DEV	7
n/a	A.28	N501T H655Y	DEV	2
n/a	C.16	L452R D614G	DEV	2
n/a	B.1.351+P384L	D80A D215G K417N E484K N501Y D614G A701V P384L	DEV	8
n/a	B.1.351+E516Q	D80A D215G K417N E484K N501Y D614G A701V E516Q	DEV	8
n/a	B.1.1.7+L452R	N501Y A570D D614G P681H T716I S982A D1118H L452R	DEV	8
n/a	B.1.1.7+S494P	N501Y A570D D614G P681H T716I S982A D1118H S494P	DEV	8
Iota	B.1.526	L5F T95I D253G D614G	DEV	4
Zeta	P.2	E484K D614G V1176F	DEV	3
n/a	B.1.1.519	T478K D614G P681H T732A	DEV	4
n/a	AV.1	D80G T95I G142D N439K E484K D614G P681H I1130V D1139H	DEV	9
n/a	AT.1	P9L D215G H245P E484K D614G E780K	DEV	6
n/a	C.36+L452R	D614G Q677H L452R	DEV	3
Mu	B.1.621	T95I Y145N R346K E484K N501Y D614G P681H D950N	DEV	8
Lambda	C.37	G75V T76I R246N L452Q F490S D614G T859N	DEV	7
n/a	AY.4.2	T19R T95I G142D Y145H E156G A222V L452R T478K D614G P681R D950N	DEV	11
n/a	B.1.1.318	T95I E484K D614G P681H D796H	DEV	5
n/a	C.1.2	P9L C136F R190S D215G Y449H E484K N501Y D614G H655Y N679K T716I T859N	DEV	12
Beta	B.1.351	D80A D215G K417N E484K N501Y D614G A701V	DEV	7
Gamma	P.1	L18F T20N P26S D138Y R190S K417T E484K N501Y D614G H655Y T1027I V1176F	DEV	12
Delta	B.1.617.2	T19R E156G L452R T478K D614G P681R D950N	DEV	7

VOC: variants of concern; DEV: de-escalated variants.

## Data Availability

The source code and data used in analysis are available at https://github.com/XDcat/PCCN, accessed on 16 January 2023.

## Supplementary Materials

The following supporting information can be downloaded at: https://www.mdpi.com/article/10.3390/ijms24043255/s1.
