# Supplementary material for: A Protein Co-Conservation Network Model Characterizes Mutation Effects on SARS-CoV-2 Spike Protein
Source: Int J Mol Sci. 2023 Feb 7;24(4):3255. doi: 10.3390/ijms24043255 (PMC9960056; doi:10.3390/ijms24043255)
Supplement: Supplementary file 1 [file ijms-24-03255-s001.zip › SM.pdf]

Supplementary Material

A protein co-conservation network model characterizes mutations effects on SARS-CoV-2 spike protein

Lianjie Zeng [1,2], Yitan Lu [3], Wenying Yan [3,4,*] and Yang Yang [1,2,*]

[1] School of Computer Science & Technology, Soochow University, Suzhou 215000, China
[2] Collaborative Innovation Center of Novel Software Technology and Industrialization, Nanjing 210000, China;
[3] Department of Bioinformatics, School of Biology and Basic Medical Sciences, Medical College of Soochow University, Suzhou 215123, China
[4] Jiangsu Province Engineering Research Center of Precision Diagnostics and Therapeutics Development, Suzhou 215123, China
* Correspondence: wyyan@suda.edu.cn (W.Y.); yyang@suda.edu.cn (Y.Y.)

Table S1 The network parameters for each node in PCCN of spike protein

See the file “TableS1.xlsx”.

Table S2 Correlation of Stability changes of spike protein upon mutations with PCCN topological features

**The stability changes upon mutations or co-mutations sites with top three topological features**	**Topological features**	**Correlation**	**P value**
G142D (-4.78), E156G (-3.67), C136F (-3.17)	*C*	0.146	0.146
	Kw	**0.253**	**0.011**
	D	**0.242**	**0.015**
	P	**0.271**	**0.006**
G142D-E156G (-4.22), G142D-Y145H (-3.62), G142D-N439K (-3.38)	*L*	-0.029	0.350
T95I-G142D (-2.98), G142D-N501Y (-2.88), P9L-T859N (-2.87)	*CCS*	0.109	0.094

Table S3 Correlation of binding free energy (BFE) changes of spike protein upon mutations with PCCN topological features

**The BFE changes upon mutations or co-mutations sites with top three topological features**	**Topological features**	**Correlation**	**P value**
Q493R(8.23), D215G (8.10), T547K(8.07)	***C***	**-0.207**	**0.045**
	Kw	-0.050	0.633
	D	-0.056	0.595
	P	0.048	0.649
Q493R-T547K(8.15),T478K-Q493R(8.13),V213G-Q493R(8.12)	***L***	**0.179**	**0.000**
D215G-D614G(8.05),S371F-D614G(7.99),D614G-T732A(7.97)	***CCS***	**0.233**	**0.001**

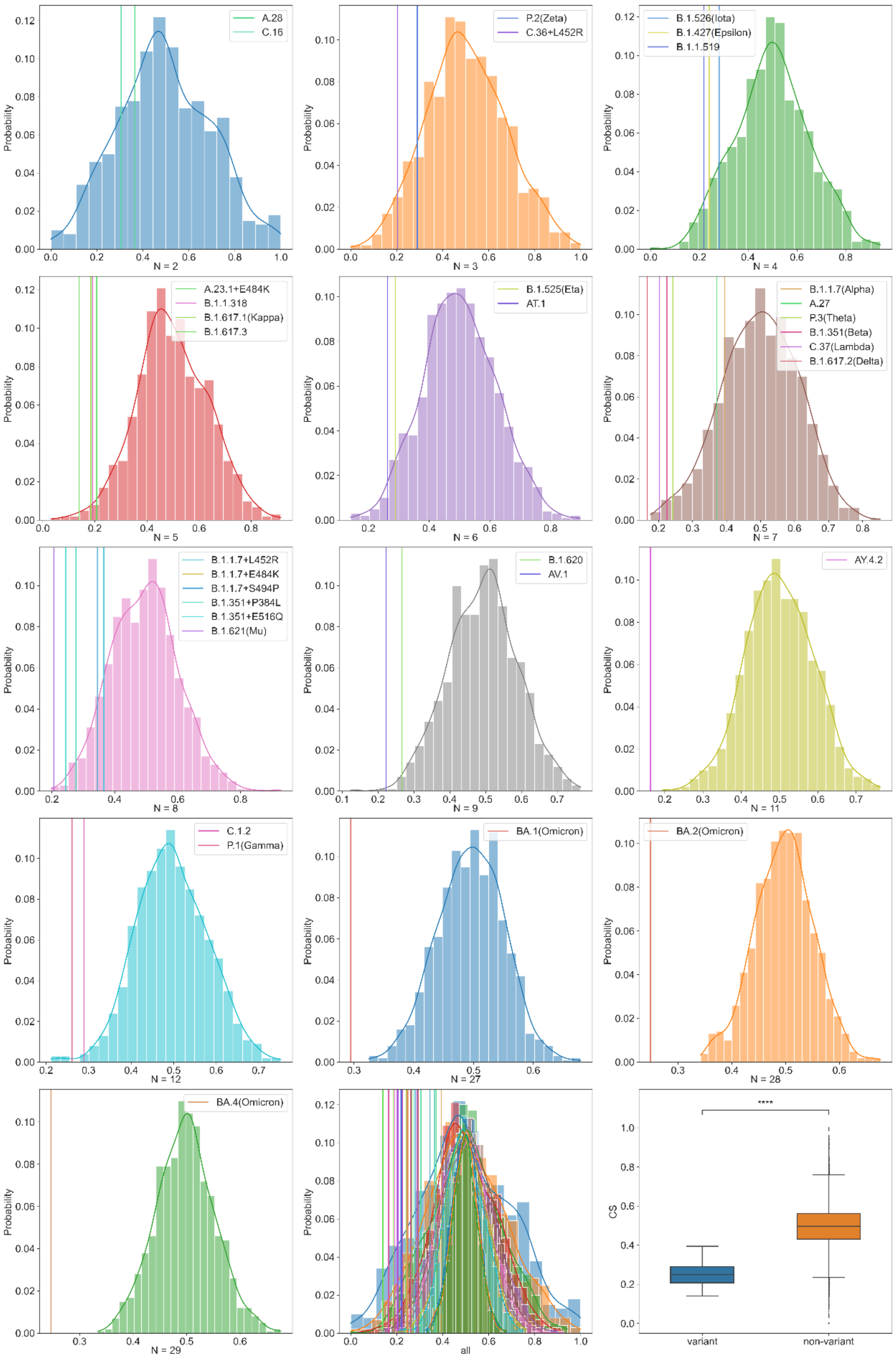

Figure S1. The distribution of CS for the same number of nodes in PCCN.

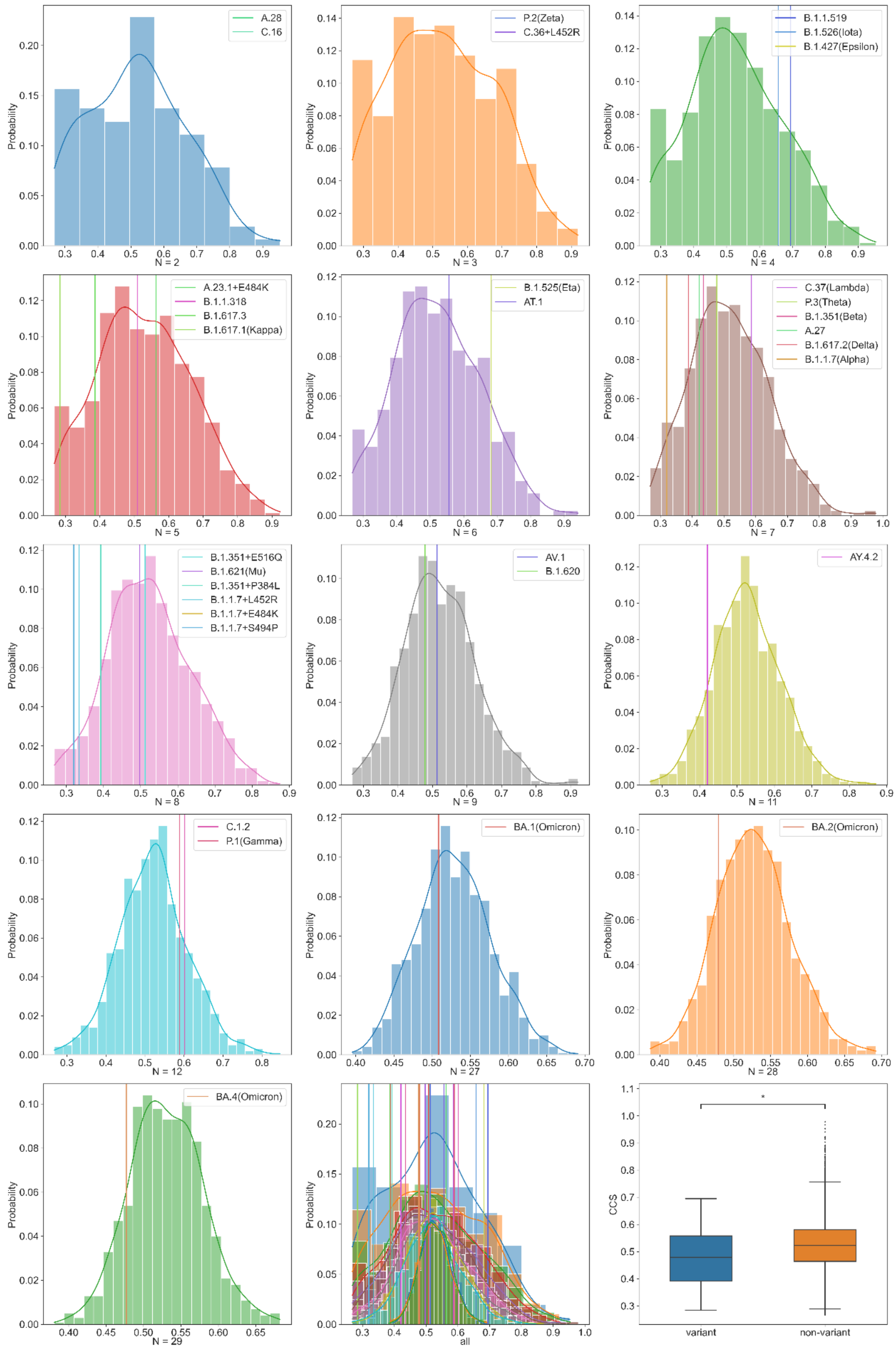

Figure S2. The distribution of CCS for the same number of nodes in PCCN.

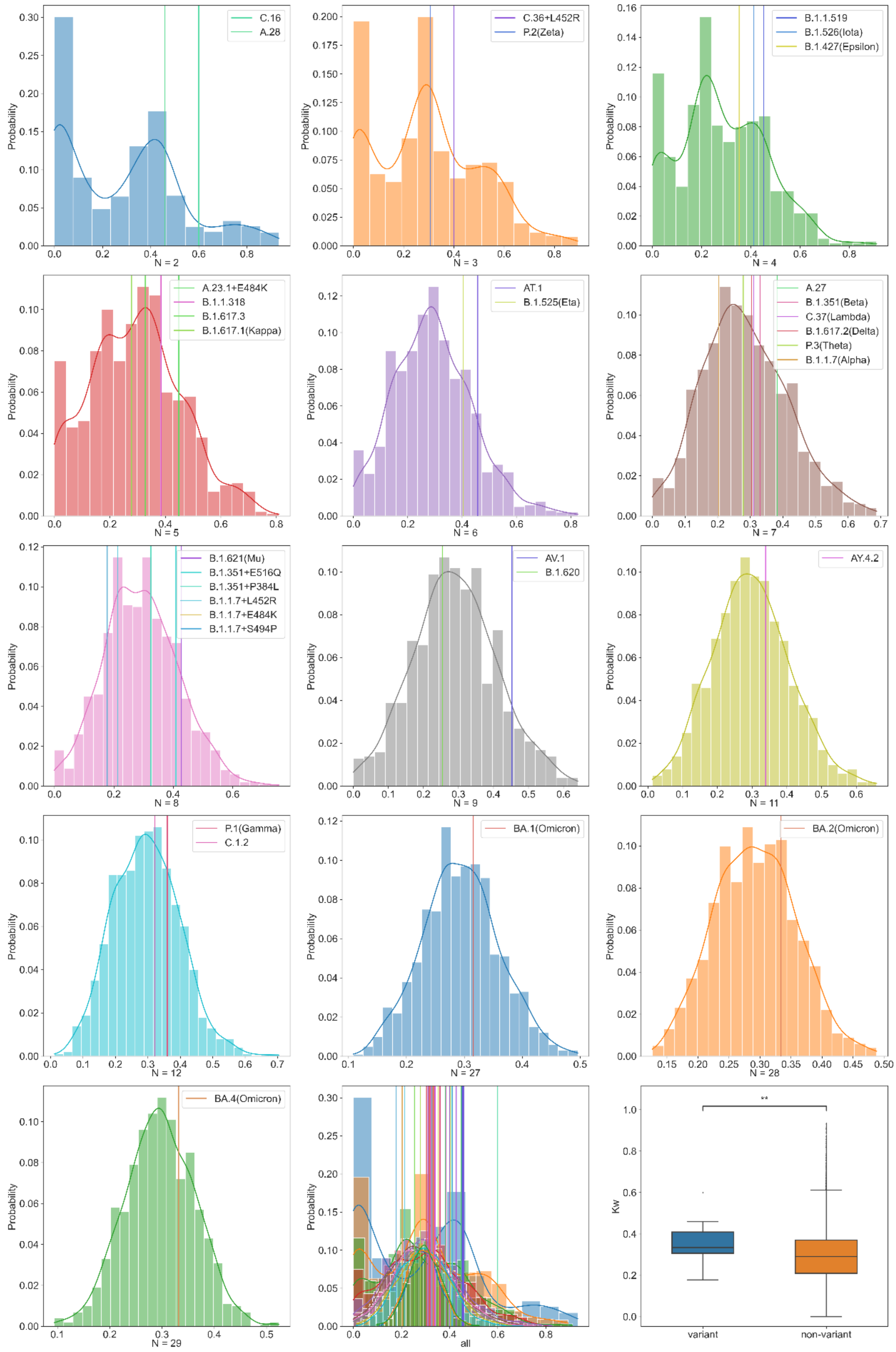

Figure S3. The distribution of Kw for the same number of nodes in PCCN.

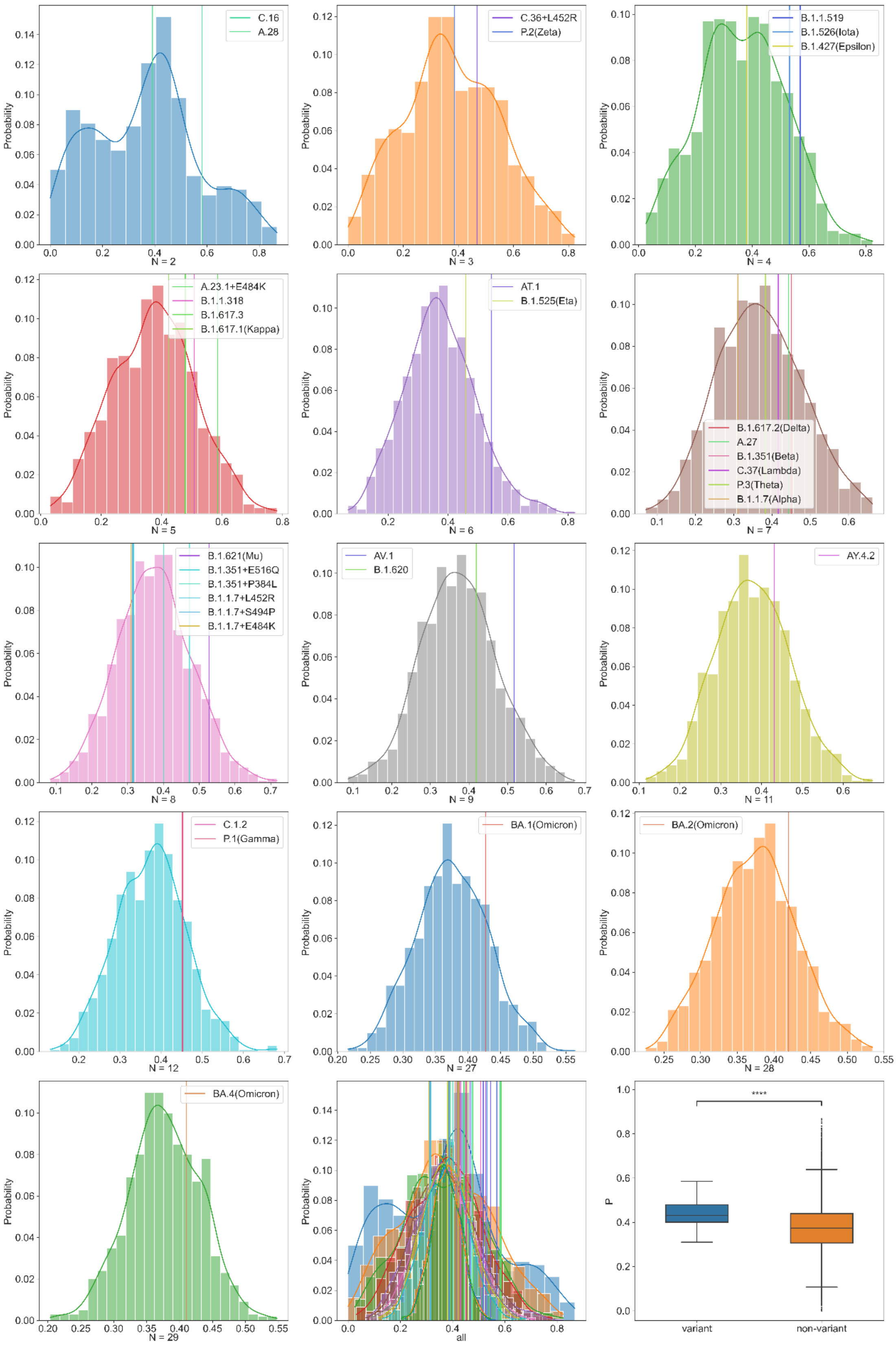

Figure S4. The distribution of P for the same number of nodes in PCCN.

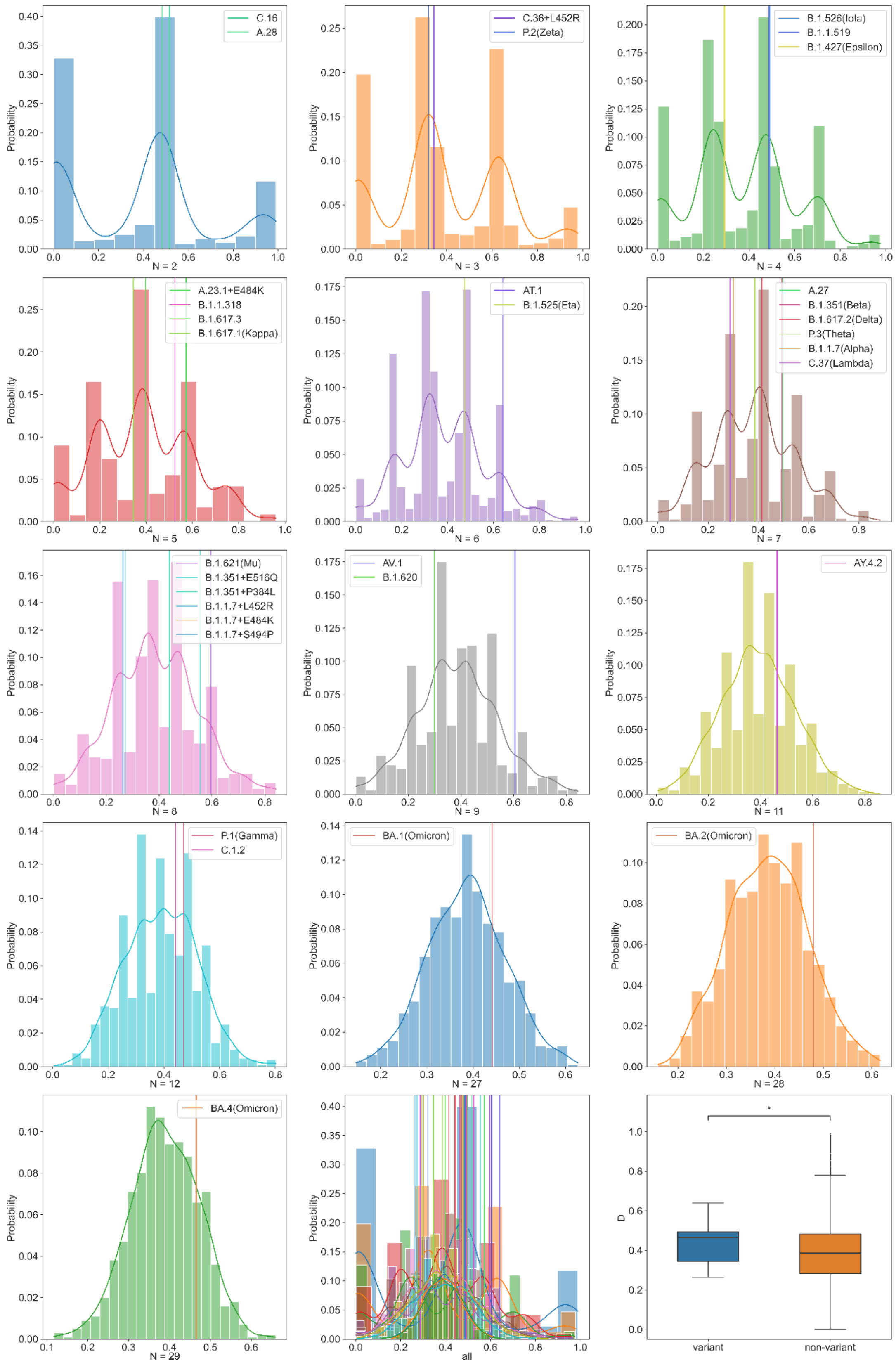

Figure S5. The distribution of D for the same number of nodes in PCCN.

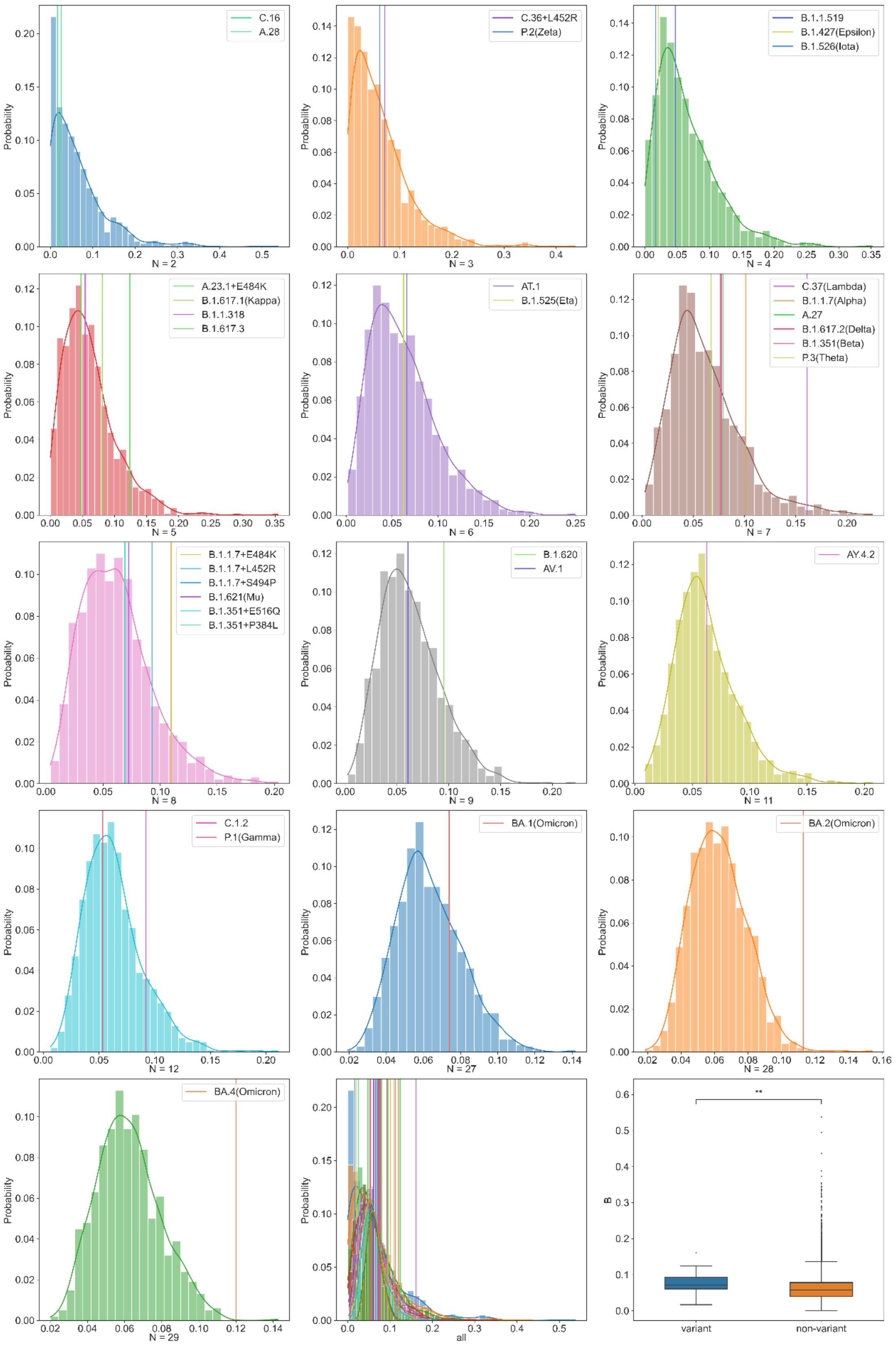

Figure S6. The distribution of B for the same number of nodes in PCCN.

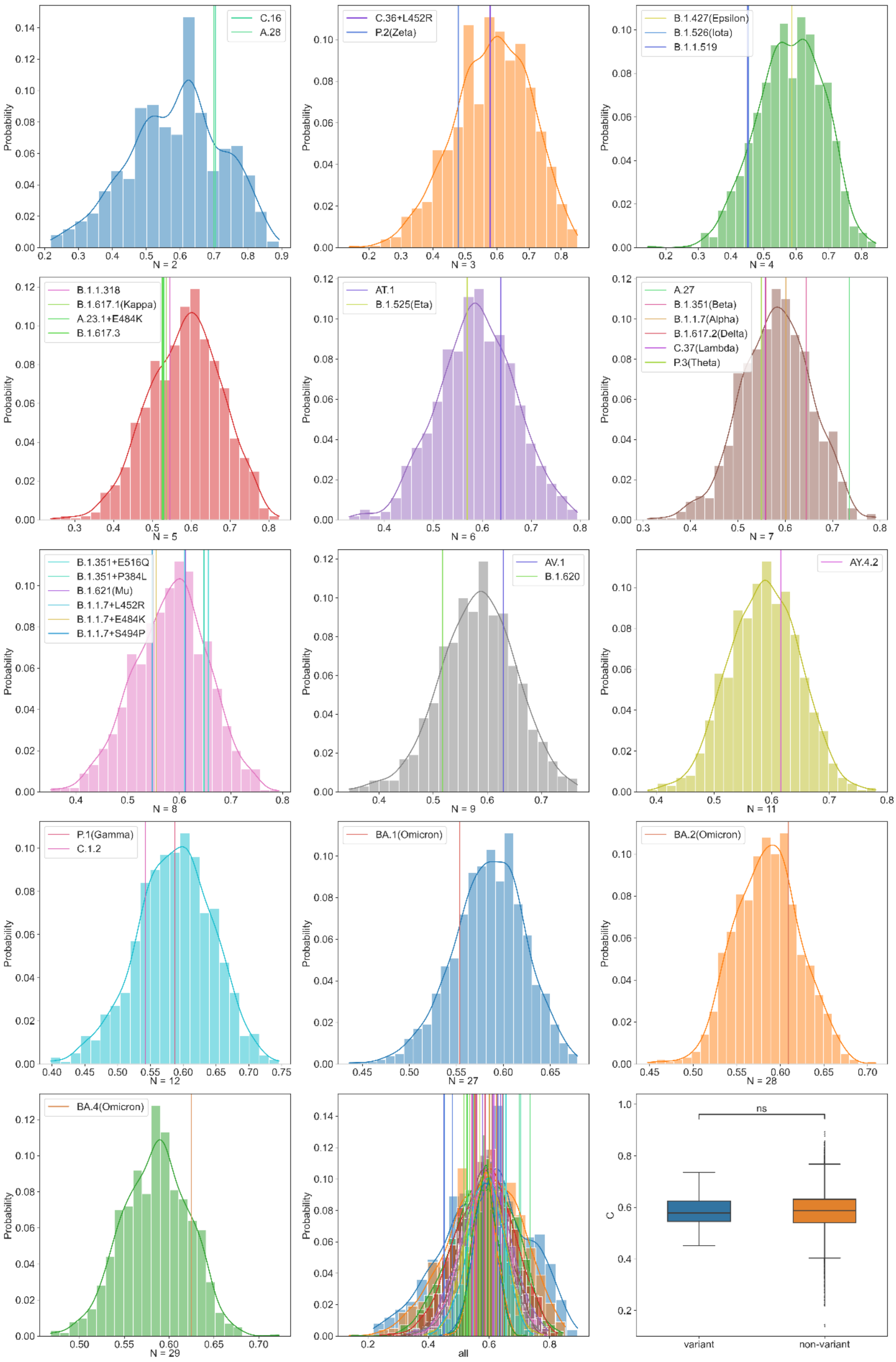

Figure S7. The distribution of C for the same number of nodes in PCCN.

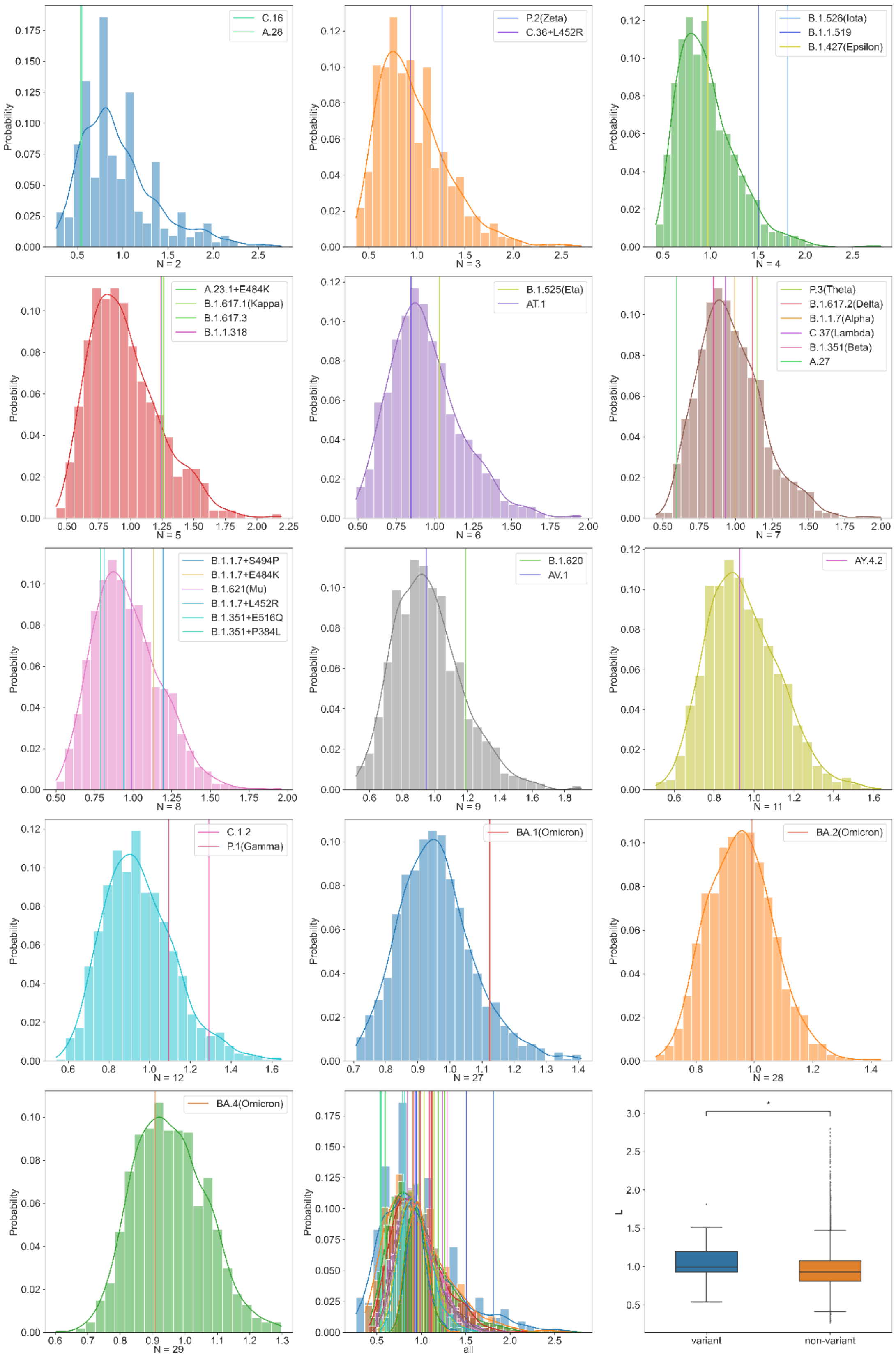

Figure S8. The distribution of L for the same number of nodes in PCCN.
